# Rubidium penta­aqua­(l-serine)cobalt(II) hexa­hydrogenhexa­molybdocobaltate(III) l-serine monosolvate deca­hydrate

**DOI:** 10.1107/S1600536813028304

**Published:** 2013-10-19

**Authors:** Jun Iijima, Haruo Naruke, Hiroshi Takiyama

**Affiliations:** a Department of Chemical Engineering, School of Engineering, Tokyo University of Agriculture and Technology, 2-24-16, Naka-cho, Koganei-city, Tokyo, Japan; b Chemical Resouces Laboratory, Tokyo Institute of Technology, 4259, Nagatsuta, Midori-ku, Yokohama-city, Kanagawa, Japan; c Division of Applied Chemistry, Institute of Engineering, Tokyo University of Agriculture and Technology, 2-24-16, Naka-cho, Koganei-city, Tokyo, Japan

## Abstract

The Co^2+^ ion in the title compound, Rb[Co(C_3_H_7_NO_3_)(H_2_O)_5_][H_6_CoMo_6_O_24_]·C_3_H_7_NO_3_·10H_2_O, is coordinated by five water mol­ecules and one *O*-monodentate l-serine ligand in a slightly distorted octahedral geometry. The Rb^+^ ion is irregularly coordinated by nine O atoms. In the crystal, the [H_6_Co^III^Mo_6_O_24_]^3−^ polyanions are stacked along the *b*-axis direction, mediated by bridging Rb—O bonds. N—H⋯O and O—H⋯O hydrogen bonds are observed involving the l-serine mol­ecules.

## Related literature

For background to polyoxidometallates (POMs), see: Hasenknopf *et al.* (2008 ▶); Du *et al.* (2013 ▶); Fang *et al.* (2005 ▶); Kortz *et al.* (2002 ▶); Sadakane *et al.* (2001 ▶); Tan *et al.* (2007 ▶); Inoue & Yamase (1995 ▶). For C—O bond lengths in carboxyl­ates, see: Lide (2007 ▶). For bond-valence sums, see: Brown (1980 ▶). For protonation of POMs, see: Perloff (1970 ▶); Honda *et al.* (2007 ▶); Yang *et al.* (2013 ▶). For chiral POMs constructed from an Anderson-type POM as a building block, see: An *et al.* (2008 ▶).
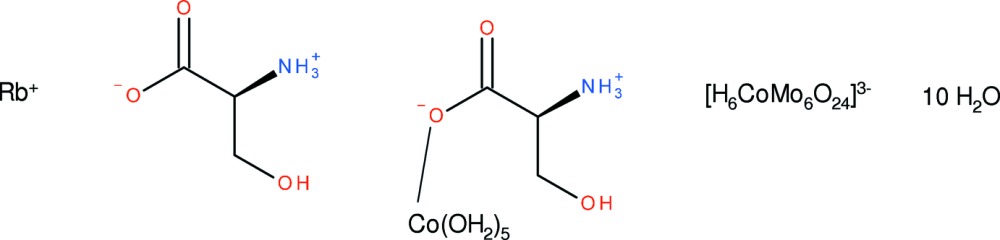


## Experimental

### 

#### Crystal data

Rb[Co(C_3_H_7_NO_3_)(H_2_O)_5_][H_6_CoMo_6_O_24_]·C_3_H_7_NO_3_·10H_2_O
*M* *_r_* = 1649.42
Orthorhombic, 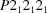

*a* = 10.8411 (5) Å
*b* = 11.5923 (4) Å
*c* = 34.8078 (12) Å
*V* = 4374.4 (3) Å^3^
*Z* = 4
Mo *K*α radiation
μ = 3.63 mm^−1^
*T* = 183 K
0.41 × 0.36 × 0.19 mm

#### Data collection

Rigaku R-AXIS RAPID diffractometer
Absorption correction: numerical (*NUMABS*; Rigaku, 1999 ▶) *T*_min_ = 0.123, *T*_max_ = 0.500
70065 measured reflections
10010 independent reflections
9728 reflections with *F* ^2^ > 2σ(*F* ^2^)
*R*_int_ = 0.062

#### Refinement

*R*[*F* ^2^ > 2σ(*F* ^2^)] = 0.036
*wR*(*F* ^2^) = 0.099
*S* = 1.04
10010 reflections
559 parameters
H-atom parameters constrained
Δρ_max_ = 3.61 e Å^−3^
Δρ_min_ = −1.10 e Å^−3^
Absolute structure: Flack (1983 ▶), 4426 Friedel pairs
Absolute structure parameter: 0.025 (7)

### 

Data collection: *RAPID-AUTO* (Rigaku/MSC, 2002 ▶); cell refinement: *RAPID-AUTO*; data reduction: *RAPID-AUTO*; program(s) used to solve structure: *SHELXS97* (Sheldrick, 2008 ▶); program(s) used to refine structure: *SHELXL97* (Sheldrick, 2008 ▶); molecular graphics: *CrystalStructure* (Rigaku, 2010 ▶); software used to prepare material for publication: *CrystalStructure*.

## Supplementary Material

Crystal structure: contains datablock(s) General, I. DOI: 10.1107/S1600536813028304/hb7134sup1.cif

Structure factors: contains datablock(s) I. DOI: 10.1107/S1600536813028304/hb7134Isup2.hkl

Additional supplementary materials:  crystallographic information; 3D view; checkCIF report

## Figures and Tables

**Table 1:** Hydrogen-bond geometry (Å, °)

*D*—H⋯*A*	*D*—H	H⋯*A*	*D*⋯*A*	*D*—H⋯*A*
O27—H4⋯O41	0.84	2.24	2.859 (6)	131
O27—H4⋯N1	0.84	2.62	2.940 (6)	104
O30—H11⋯O42^i^	0.84	2.24	2.928 (6)	139
N1—H5⋯O2	0.91	2.11	2.944 (6)	151
N1—H6⋯O39^i^	0.91	2.14	3.041 (6)	170
N1—H7⋯O17^ii^	0.91	2.09	2.905 (6)	149
N2—H13⋯O43	0.91	2.11	3.013 (7)	170
N2—H12⋯O8^iii^	0.91	2.31	2.912 (6)	123
N2—H14⋯O13^iv^	0.91	1.93	2.827 (6)	167

Symmetry codes: (i) 
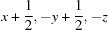
; (ii) 
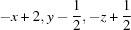
; (iii) 
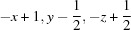
; (iv) 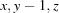.

**Fig. 1.**
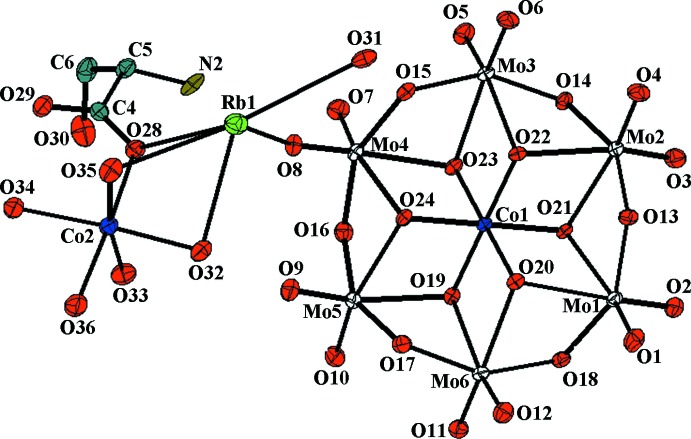
The asymmetric unit of the title compound with displacement ellipsoids drawn at the 50% probability level. White, red, blue, green, moss green and yellowish brown ellipsoids denote Mo, O, Co, Rb, C, and N atoms, respectively. The H atoms protonated to the CoMo6 polyanion are omitted for clarity.

**Fig. 2.**
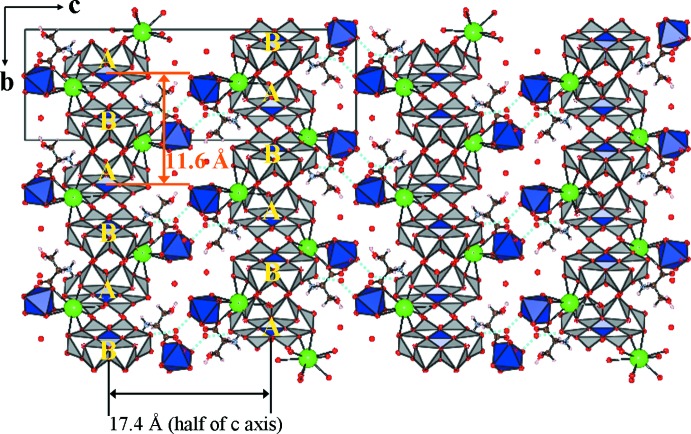
Packing diagram viewed along the a axis. The rectangle traced with standard line indicates the unit cell. Color codes; white octahedron: MoO6, blue octahedron CoO6, and Co(II) complex, green sphere: Rb, red sphere O. The hydrogen bondings are denoted by sky blue dashed lines. The L-Ser molecule and waters of crystallization O atoms unrelated to hydrogen bonding are omitted for clarity.

**Fig. 3.**
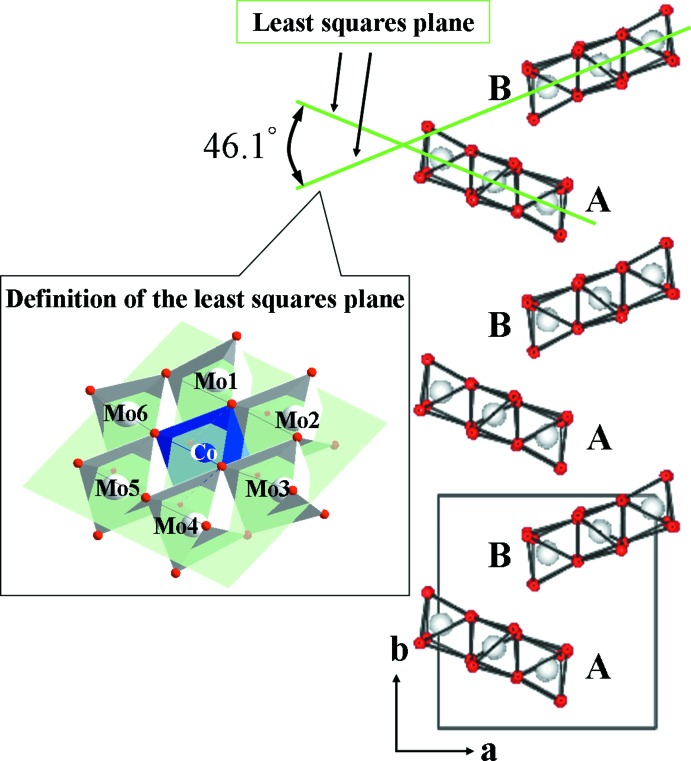
The alternate polyanion stacking of A-type and B-type viewed along the c axis. The square shows the unit cell. The MoO6 octahedron is denoted in open faced manner, and the O atoms in the CoMo6 polyanion are denoted by red spheres. The definition of the least square plane consisting of Co, Mo1, Mo2, Mo3, Mo4, Mo5, and Mo6 in the CoMo6 polyanion is also represented.

## supplementary crystallographic information

### 1. Comment

Continuous interests in chiral polyoxometalates (POMs) are endowed owing to their prominent molecular applications such as nonlinear optics, magneto optical effect, and circular polarized luminescence (Hasenknopf *et al.*, 2008; Du *et al.*, 2013). Two synthetic procedures for chiral POMs have been delivered to date. One is the way based on the connection of chiral organic ligands and achiral inorganic POMs possible to propagate the local chirality to POM framework, which have been extensively studied by the groups of Hill, Kortz, Pope, Wang, and Yamase for a long time (Fang *et al.*, 2005; Kortz *et al.*, 2002; Sadakane *et al.*, 2001; Tan *et al.*, 2007, Inoue & Yamase, 1995). Although Anderson type POM is one of the typical achiral structures along with Keggin and Wells-Dawson types, there are little chiral POMs constructed from Anderson type POM as building block (An *et al.*, 2008). Herein, the structural characterization of the chiral crystal containing Anderson type [H_6_CoMo_6_O_24_]^3-^ as building block and amino acid *L*-Serine (*L*-Ser) as chiral organic ligand is reported. The crystallization of enantiomeric unit (Figure 1) consisting of the CoMo6 polyanion, [Co(H_2_O)_5_(*L*-Ser)]^2+^ (Co(II) complex), Rb^+^, one water molecule, and free *L*-Ser, and the other water molecules with noncentrosymmetric space group *P*2_1_2_1_2_1_ provided the title compound, of which the absolute configuration was supported by Flack parameter 0.025 (7). The p*K*_a_ (2.2 and 9.2) of *L*-Ser and the acidic condition (pH 3.2) in the synthetic procedure suggest that all *L*-Ser molecules are zwitterionic. In fact, the carboxylate group in *L*-Ser coordinating to Co^2+^ and that in free *L*-Ser have similar C—O distances (1.250 (7), 1.264 (7) Å and 1.247 (7), 1.257 (7) Å) due to their resonance state (Lide, 2007). The BVS values calculated from the observed bond lengths are 2.0 and 3.2 for Co in Co(II) complex and the CoMo6 polyanion, 5.9–6.0 for Mo, indicating that the original valences of Co^2+^, Co^3+^, and Mo^6+^ are retained in the title compound. Additionally, the BVS values for six O atoms forming CoO_6_ octahedra in the CoMo6 polyanion are 1.1–1.2, suggesting that they are protonated (Brown, 1980). Similar protonation in Anderson type POMs have been frequently observed for [H_6_CrMo_6_O_24_]^3-^, [H_2_IMo_6_O_24_]^3-^, and [H_6_NiMo_6_O_24_]^4-^ (Perloff, 1970; Honda *et al.*, 2007; Yang *et al.*, 2013). Figure 2 shows the crystal structure viewed along the *a* axis. The two kinds of polyanion (A-type and B-type) with different orientation in the *ac* plane are alternately connected along the crystallographic *b* axis by Rb^+^-bridging of terminal O atoms in the CoMo6 polyanion, forming the b-axially stacked polyanion unit. The polyanion units are aligned along the *c* axis with a help of hydrogen bonding (O···O < 3.3 Å) relating with some waters of crystallization and aqua ligands in Co(II) complex, of which the unit distance is the half of *c* axis value. The both distance of A-type···A-type and B-type···B-type are 11.6 Å; (Co···Co distance), which corresponds to the *b* axis value. Figure 3 is the representation of the b-axially polyanion stacking mentioned above along the *c* axis. It is clearly demonstrated that A-type polyanion and B-type polyanion are altenately stacked with a cross angle of 46.1°, which is calculated from the dihedral angle of the least square plane defined by Co, Mo1, Mo2, Mo3, Mo4, Mo5, and Mo6 in the CoMo6 polyanion.

### 2. Experimental

An aqueous solution containing the Na salt of CoMo6, *L*-Ser, and CoCl_2_·6H_2_O with a molar ratio of 1:4:2 was acidified to pH = 3.2 by conc HCl and then boiled for 1 h. After cooling to room temperature, 5 eq. of RbClO_4_ to CoMo6 was added into the solution. Viridian thin platelets of the title compound were obtained at 4 °C being stood for several days.

### 3. Refinement

Fragile thin platelet crystals of the title compound gave a medium absorption corrections (transmission factor ranging from 12.3 to 50.0%), resulting in a somewhat high residual electron densities around Mo1, Mo2, Mo4, and Mo6. Generally, a complete convergence of structure refinement with residual density less than 1 e Å^-3^ in difference Fourier synthesis indicates the success of structural analysis. However, for the compounds containing many heavy atoms, since the anisotropy of electron cloud around the heavy atoms makes difficult to conduct the ellipse approximation, the relative large residual densities cannot help being remaining. In the structural analysis of polyoxometalate consisting of a variety of heavy atoms such as Mo, V, and W, the relatively high residual densities have been frequently observed for the crystals with poor absorption corrections and crystallinity. In fact, after some elements were allocated to the pointed out densities, the refinements resulted in the divergence. Therefore, the pointed out high residual densities for Mo1, Mo2, Mo4, and Mo6 are not responsible for the incompleteness of structural analysis but the intrinsic crystal qualities such as morphology and crystallinity. All the atoms except for H atoms were refined anisotropically, and the H atoms were isotropically. The H atoms in *L*-Ser molecules were located in the calculated position, and the remaining H atoms were not included in the refinements. The residual density around O31 may be H atom. Due to the undesirable absorption correction, it is conceivable that high residual density corresponding to H atom is remained. In the polyoxometalate bearing many heavy atoms, the electron density for H atom is hard to be found. In order to escape from the divergence and lowering the accuracy of refinement caused by intentional assignment of H atom to remarkable low electron density, for polyoxometalate compounds, the H atoms in water crystallization are not included in refinements. Therefore, the water of crystallization O atoms (O37 to O45) are refined without H atoms.

### Crystal data

**Table:**

Rb[Co(C_3_H_7_NO_3_)(H_2_O)_5_][H_6_CoMo_6_O_24_]·C_3_H_7_NO_3_·10H_2_O	*F*(000) = 3212.00
*M**_r_* = 1649.42	*D*_x_ = 2.504 Mg m^−^^3^
Orthorhombic, *P*2_1_2_1_2_1_	Mo *K*α radiation, λ = 0.71075 Å
Hall symbol: P 2ac 2ab	Cell parameters from 59700 reflections
*a* = 10.8411 (5) Å	θ = 3.0–27.5°
*b* = 11.5923 (4) Å	µ = 3.63 mm^−^^1^
*c* = 34.8078 (12) Å	*T* = 183 K
*V* = 4374.4 (3) Å^3^	Platelet, green
*Z* = 4	0.41 × 0.36 × 0.19 mm

### Data collection

**Table:**

Rigaku R-AXIS RAPID diffractometer	9728 reflections with *F*^2^ > 2σ(*F*^2^)
Detector resolution: 10.000 pixels mm^-1^	*R*_int_ = 0.062
ω scans	θ_max_ = 27.5°
Absorption correction: numerical (*NUMABS*; Rigaku, 1999)	*h* = −14→14
*T*_min_ = 0.123, *T*_max_ = 0.500	*k* = −15→14
70065 measured reflections	*l* = −45→45
10010 independent reflections	

### Refinement

**Table:**

Refinement on *F*^2^	Hydrogen site location: inferred from neighbouring sites
*R*[*F*^2^ > 2σ(*F*^2^)] = 0.036	H-atom parameters constrained
*wR*(*F*^2^) = 0.099	*w* = 1/[σ^2^(*F*_o_^2^) + (0.0641*P*)^2^ + 9.0772*P*] where *P* = (*F*_o_^2^ + 2*F*_c_^2^)/3
*S* = 1.04	(Δ/σ)_max_ = 0.001
10010 reflections	Δρ_max_ = 3.61 e Å^−^^3^
559 parameters	Δρ_min_ = −1.10 e Å^−^^3^
0 restraints	Absolute structure: Flack (1983), 4426 Friedel pairs
Primary atom site location: structure-invariant direct methods	Absolute structure parameter: 0.025 (7)
Secondary atom site location: difference Fourier map	

### Special details

**Table:**

Geometry. ENTER SPECIAL DETAILS OF THE MOLECULAR GEOMETRY
Refinement. Refinement was performed using all reflections. The weighted *R*-factor (*wR*) and goodness of fit (*S*) are based on *F*^2^. *R*-factor (gt) are based on *F*. The threshold expression of *F*^2^ > 2.0 σ(*F*^2^) is used only for calculating *R*-factor (gt).

### Fractional atomic coordinates and isotropic or equivalent isotropic displacement parameters (Å^2^)

**Table:**

	*x*	*y*	*z*	*U*_iso_*/*U*_eq_	
Mo1	0.98964 (4)	0.94684 (3)	0.207793 (11)	0.01204 (9)	
Mo2	0.74633 (3)	0.84989 (3)	0.159456 (12)	0.01213 (9)	
Mo3	0.49911 (3)	0.75710 (3)	0.205914 (11)	0.01245 (9)	
Mo4	0.50288 (4)	0.74903 (3)	0.301413 (11)	0.01297 (9)	
Mo5	0.74159 (3)	0.84414 (3)	0.349594 (12)	0.01364 (9)	
Mo6	0.98220 (4)	0.94607 (3)	0.303320 (11)	0.01208 (9)	
Rb1	0.73248 (5)	0.52247 (4)	0.145507 (15)	0.02399 (11)	
Co1	0.74451 (5)	0.84817 (4)	0.254347 (19)	0.00969 (12)	
Co2	0.67086 (6)	0.46834 (6)	0.043914 (18)	0.01672 (14)	
O1	1.0518 (4)	1.0822 (4)	0.20918 (11)	0.0217 (8)	
O2	1.0846 (4)	0.8663 (4)	0.17903 (10)	0.0212 (8)	
O3	0.8426 (4)	0.7691 (3)	0.13123 (10)	0.0196 (7)	
O4	0.6534 (4)	0.9266 (4)	0.12866 (10)	0.0203 (8)	
O5	0.4363 (4)	0.6206 (3)	0.20593 (11)	0.0205 (8)	
O6	0.4087 (4)	0.8391 (4)	0.17621 (10)	0.0205 (8)	
O7	0.4401 (4)	0.6137 (3)	0.29822 (11)	0.0211 (8)	
O8	0.4089 (4)	0.8259 (3)	0.33182 (10)	0.0181 (8)	
O9	0.6422 (4)	0.9134 (4)	0.37969 (11)	0.0233 (8)	
O10	0.8398 (4)	0.7659 (4)	0.37805 (10)	0.0229 (8)	
O11	1.0744 (3)	0.8673 (3)	0.33354 (10)	0.0174 (7)	
O12	1.0443 (4)	1.0825 (3)	0.30257 (11)	0.0200 (8)	
O13	0.8528 (3)	0.9780 (3)	0.17385 (9)	0.0140 (7)	
O14	0.6360 (3)	0.7246 (3)	0.17290 (10)	0.0146 (7)	
O15	0.4347 (3)	0.8152 (3)	0.25403 (10)	0.0156 (7)	
O16	0.6406 (4)	0.7154 (3)	0.33395 (10)	0.0172 (7)	
O17	0.8416 (3)	0.9759 (3)	0.33605 (10)	0.0166 (7)	
O18	1.0544 (3)	0.8863 (3)	0.25617 (10)	0.0143 (7)	
O19	0.8487 (3)	0.7967 (3)	0.29521 (9)	0.0122 (7)	
O20	0.8484 (3)	0.9818 (3)	0.25489 (9)	0.0120 (6)	
O21	0.8514 (3)	0.7968 (3)	0.21394 (9)	0.0109 (7)	
O22	0.6412 (3)	0.9013 (3)	0.21348 (9)	0.0117 (7)	
O23	0.6387 (3)	0.7160 (3)	0.25320 (10)	0.0130 (6)	
O24	0.6384 (3)	0.8976 (3)	0.29495 (9)	0.0124 (7)	
O25	1.0441 (4)	0.5935 (4)	0.04817 (12)	0.0314 (10)	
O26	1.2444 (4)	0.5865 (4)	0.06457 (11)	0.0256 (8)	
O27	1.1876 (4)	0.8681 (4)	0.06784 (11)	0.0254 (8)	
O28	0.7536 (3)	0.3482 (3)	0.08204 (10)	0.0184 (7)	
O29	0.9125 (4)	0.2885 (4)	0.04575 (11)	0.0243 (8)	
O30	0.7725 (4)	0.0466 (4)	0.05742 (11)	0.0334 (10)	
O31	0.7102 (3)	0.5053 (3)	0.23535 (11)	0.0208 (8)	
O32	0.5473 (4)	0.5398 (4)	0.08612 (11)	0.0229 (8)	
O33	0.5412 (4)	0.3362 (4)	0.03548 (11)	0.0247 (8)	
O34	0.7917 (4)	0.4190 (4)	−0.00155 (10)	0.0216 (8)	
O35	0.7958 (4)	0.5901 (4)	0.06412 (11)	0.0243 (8)	
O36	0.5870 (4)	0.5650 (4)	0.00168 (11)	0.0316 (10)	
O37	0.6585 (4)	0.2721 (4)	−0.04481 (11)	0.0275 (9)	
O38	0.2036 (4)	0.6949 (4)	0.2567 (2)	0.0565 (18)	
O39	0.9618 (4)	−0.2655 (4)	−0.09497 (11)	0.0265 (9)	
O40	0.8044 (4)	0.0929 (4)	−0.01818 (11)	0.0250 (9)	
O41	1.3218 (5)	1.0091 (4)	0.12069 (12)	0.0361 (10)	
O42	0.2127 (4)	0.6982 (4)	−0.04824 (14)	0.0353 (10)	
O43	0.5376 (5)	0.1397 (4)	0.08536 (14)	0.0382 (11)	
O44	0.3835 (4)	0.6982 (4)	0.01085 (12)	0.0326 (10)	
O45	0.4649 (5)	0.9210 (4)	0.02937 (13)	0.0389 (11)	
N1	1.2151 (5)	0.6988 (4)	0.13015 (12)	0.0201 (9)	
N2	0.7690 (4)	0.1719 (4)	0.13211 (14)	0.0199 (9)	
C1	1.1344 (5)	0.6185 (5)	0.06875 (15)	0.0201 (10)	
C2	1.1089 (5)	0.7005 (5)	0.10294 (14)	0.0178 (10)	
C3	1.0849 (6)	0.8225 (5)	0.08854 (16)	0.0240 (11)	
C4	0.8428 (5)	0.2819 (5)	0.07428 (13)	0.0170 (10)	
C5	0.8689 (5)	0.1813 (5)	0.10152 (15)	0.0193 (10)	
C6	0.8809 (6)	0.0683 (5)	0.07916 (16)	0.0253 (12)	
H1	1.0337	0.6727	0.1168	0.0214*	
H2	1.0115	0.8221	0.0716	0.0288*	
H13	0.6941	0.1642	0.1206	0.0238*	
H12	0.7693	0.2367	0.1469	0.0238*	
H14	0.7839	0.1092	0.1472	0.0238*	
H3	1.0668	0.8732	0.1107	0.0288*	
H4	1.2497	0.8700	0.0823	0.0305*	
H5	1.1981	0.7449	0.1506	0.0241*	
H6	1.2839	0.7251	0.1180	0.0241*	
H7	1.2282	0.6254	0.1384	0.0241*	
H8	0.9492	0.1966	0.1147	0.0231*	
H9	0.9528	0.0728	0.0617	0.0304*	
H10	0.8950	0.0039	0.0973	0.0304*	
H11	0.7805	−0.0157	0.0453	0.0401*	

### Atomic displacement parameters (Å^2^)

**Table:**

	*U*^11^	*U*^22^	*U*^33^	*U*^12^	*U*^13^	*U*^23^
Mo1	0.00991 (17)	0.01256 (18)	0.01365 (17)	−0.00216 (14)	0.00079 (14)	0.00013 (14)
Mo2	0.01080 (18)	0.01307 (19)	0.01252 (18)	−0.00066 (15)	−0.00029 (13)	0.00023 (13)
Mo3	0.00977 (18)	0.01192 (18)	0.01566 (18)	−0.00179 (15)	−0.00060 (14)	−0.00069 (14)
Mo4	0.01106 (18)	0.01205 (18)	0.01579 (18)	−0.00148 (15)	0.00241 (15)	0.00050 (14)
Mo5	0.01245 (19)	0.01610 (19)	0.01237 (18)	0.00063 (16)	0.00120 (13)	−0.00002 (14)
Mo6	0.00996 (17)	0.01245 (18)	0.01384 (18)	−0.00125 (14)	−0.00103 (14)	−0.00076 (14)
Rb1	0.0239 (3)	0.0221 (3)	0.0259 (3)	0.0034 (2)	−0.00233 (19)	−0.0013 (2)
Co1	0.0077 (3)	0.0092 (3)	0.0121 (3)	0.0002 (2)	0.0003 (2)	−0.0001 (2)
Co2	0.0147 (3)	0.0177 (3)	0.0177 (3)	0.0013 (3)	0.0006 (3)	0.0017 (3)
O1	0.0194 (18)	0.0199 (18)	0.0257 (19)	−0.0014 (15)	0.0031 (15)	0.0068 (16)
O2	0.0160 (17)	0.029 (2)	0.0191 (18)	0.0003 (15)	0.0028 (14)	−0.0047 (16)
O3	0.0191 (18)	0.0188 (17)	0.0210 (17)	0.0008 (15)	−0.0011 (14)	−0.0049 (15)
O4	0.0196 (18)	0.0209 (17)	0.0204 (17)	−0.0006 (15)	−0.0066 (14)	0.0056 (15)
O5	0.0197 (18)	0.0162 (16)	0.0257 (19)	−0.0058 (14)	0.0034 (15)	−0.0026 (15)
O6	0.0161 (17)	0.027 (2)	0.0188 (17)	0.0060 (16)	−0.0053 (14)	0.0008 (16)
O7	0.0223 (19)	0.0164 (17)	0.0246 (19)	−0.0067 (15)	−0.0016 (16)	0.0025 (15)
O8	0.0188 (18)	0.0186 (18)	0.0170 (17)	0.0014 (14)	0.0040 (14)	0.0021 (15)
O9	0.0195 (18)	0.030 (2)	0.0208 (18)	−0.0012 (15)	0.0054 (15)	−0.0027 (16)
O10	0.0213 (18)	0.030 (2)	0.0174 (18)	0.0017 (16)	−0.0018 (15)	0.0075 (16)
O11	0.0159 (16)	0.0191 (18)	0.0174 (17)	0.0009 (14)	−0.0011 (13)	0.0013 (15)
O12	0.0177 (18)	0.0194 (17)	0.0228 (18)	0.0024 (14)	0.0017 (15)	0.0010 (15)
O13	0.0140 (15)	0.0110 (15)	0.0169 (16)	−0.0037 (13)	−0.0031 (13)	0.0025 (13)
O14	0.0139 (16)	0.0130 (15)	0.0168 (16)	−0.0049 (13)	0.0029 (13)	−0.0019 (14)
O15	0.0102 (14)	0.0157 (15)	0.0209 (17)	0.0005 (11)	0.0018 (14)	0.0014 (14)
O16	0.0189 (18)	0.0165 (17)	0.0164 (17)	0.0017 (14)	0.0010 (13)	0.0024 (14)
O17	0.0148 (16)	0.0171 (17)	0.0179 (16)	0.0012 (14)	0.0000 (13)	−0.0055 (14)
O18	0.0099 (14)	0.0153 (15)	0.0178 (16)	0.0010 (11)	−0.0008 (13)	−0.0011 (14)
O19	0.0120 (15)	0.0107 (15)	0.0139 (16)	0.0004 (12)	0.0012 (12)	0.0006 (13)
O20	0.0105 (14)	0.0095 (13)	0.0161 (15)	0.0002 (11)	0.0004 (12)	−0.0011 (13)
O21	0.0072 (14)	0.0104 (14)	0.0152 (16)	−0.0005 (11)	0.0016 (12)	−0.0014 (13)
O22	0.0095 (15)	0.0098 (15)	0.0159 (16)	−0.0007 (12)	−0.0020 (12)	0.0007 (13)
O23	0.0108 (14)	0.0098 (13)	0.0184 (16)	−0.0018 (11)	0.0014 (13)	−0.0002 (14)
O24	0.0108 (15)	0.0109 (15)	0.0154 (16)	−0.0013 (12)	0.0018 (13)	0.0016 (13)
O25	0.022 (2)	0.041 (3)	0.031 (3)	−0.0027 (17)	−0.0023 (17)	−0.0115 (19)
O26	0.0184 (18)	0.0302 (19)	0.028 (2)	0.0011 (16)	0.0004 (16)	−0.0045 (17)
O27	0.0224 (19)	0.029 (2)	0.0243 (19)	−0.0013 (16)	−0.0044 (16)	0.0040 (17)
O28	0.0178 (17)	0.0171 (17)	0.0203 (17)	0.0046 (15)	−0.0001 (13)	−0.0011 (13)
O29	0.0192 (18)	0.030 (2)	0.0237 (19)	0.0063 (15)	0.0047 (15)	0.0074 (17)
O30	0.044 (3)	0.028 (2)	0.028 (2)	−0.014 (2)	0.0039 (18)	−0.0053 (18)
O31	0.0145 (16)	0.0127 (16)	0.035 (2)	0.0019 (13)	−0.0039 (14)	−0.0001 (15)
O32	0.0158 (16)	0.0253 (19)	0.0276 (19)	0.0024 (15)	0.0028 (14)	−0.0021 (16)
O33	0.0200 (18)	0.0232 (18)	0.031 (2)	−0.0027 (15)	−0.0017 (15)	0.0007 (17)
O34	0.0203 (18)	0.0229 (17)	0.0217 (18)	0.0000 (15)	0.0020 (14)	−0.0005 (15)
O35	0.0225 (19)	0.0197 (18)	0.031 (2)	−0.0033 (14)	0.0032 (16)	−0.0035 (16)
O36	0.026 (2)	0.042 (3)	0.028 (2)	0.0136 (19)	0.0042 (16)	0.0127 (19)
O37	0.0190 (18)	0.040 (3)	0.0231 (19)	−0.0027 (17)	−0.0008 (15)	−0.0048 (18)
O38	0.0155 (19)	0.0092 (17)	0.145 (6)	0.0025 (14)	0.003 (3)	−0.008 (3)
O39	0.0219 (19)	0.029 (2)	0.0283 (19)	0.0050 (17)	0.0029 (15)	−0.0050 (17)
O40	0.023 (2)	0.027 (2)	0.025 (2)	−0.0047 (15)	−0.0025 (15)	−0.0065 (16)
O41	0.050 (3)	0.027 (2)	0.031 (3)	0.010 (2)	−0.007 (2)	−0.0032 (19)
O42	0.034 (3)	0.027 (2)	0.045 (3)	−0.0032 (18)	0.005 (2)	−0.0022 (19)
O43	0.034 (3)	0.033 (3)	0.047 (3)	0.0042 (19)	0.000 (2)	0.010 (2)
O44	0.028 (3)	0.037 (3)	0.032 (3)	0.0086 (18)	0.0041 (18)	0.0043 (19)
O45	0.034 (3)	0.044 (3)	0.039 (3)	0.015 (2)	0.003 (2)	0.006 (2)
N1	0.028 (3)	0.017 (2)	0.016 (2)	0.0029 (17)	−0.0006 (17)	0.0017 (17)
N2	0.0127 (19)	0.0080 (16)	0.039 (3)	0.0054 (15)	0.0093 (18)	0.0129 (18)
C1	0.019 (3)	0.021 (3)	0.020 (3)	0.003 (2)	0.0010 (19)	−0.003 (2)
C2	0.016 (3)	0.019 (3)	0.018 (3)	0.0013 (18)	0.0034 (19)	0.001 (2)
C3	0.026 (3)	0.022 (3)	0.024 (3)	0.010 (2)	0.003 (2)	0.007 (3)
C4	0.017 (3)	0.019 (3)	0.015 (2)	−0.0000 (19)	−0.0012 (18)	−0.0021 (19)
C5	0.015 (3)	0.020 (3)	0.023 (3)	0.0019 (19)	0.0035 (19)	0.006 (2)
C6	0.028 (3)	0.020 (3)	0.028 (3)	0.009 (3)	0.008 (3)	0.001 (3)

### Geometric parameters (Å, º)

**Table:**

Mo1—O1	1.709 (4)	Rb1—O28	3.002 (4)
Mo1—O2	1.713 (4)	Rb1—O31	3.143 (4)
Mo1—O13	1.931 (4)	Rb1—O32	2.889 (4)
Mo1—O18	1.955 (4)	Rb1—O35	3.018 (4)
Mo1—O20	2.280 (3)	Co1—O19	1.912 (4)
Mo1—O21	2.306 (3)	Co1—O20	1.915 (3)
Mo2—O3	1.712 (4)	Co1—O21	1.917 (4)
Mo2—O4	1.719 (4)	Co1—O22	1.913 (4)
Mo2—O13	1.946 (4)	Co1—O23	1.915 (3)
Mo2—O14	1.939 (4)	Co1—O24	1.910 (4)
Mo2—O21	2.296 (4)	Co2—O28	2.123 (4)
Mo2—O22	2.278 (4)	Co2—O32	2.154 (4)
Mo3—O5	1.722 (4)	Co2—O33	2.099 (4)
Mo3—O6	1.713 (4)	Co2—O34	2.132 (4)
Mo3—O14	1.914 (4)	Co2—O35	2.078 (4)
Mo3—O15	1.936 (4)	Co2—O36	2.060 (5)
Mo3—O22	2.288 (3)	O25—C1	1.248 (7)
Mo3—O23	2.286 (4)	O26—C1	1.257 (7)
Mo4—Mo5	3.2749 (6)	O27—C3	1.428 (7)
Mo4—O7	1.713 (4)	O28—C4	1.264 (6)
Mo4—O8	1.718 (4)	O29—C4	1.250 (6)
Mo4—O15	1.964 (4)	O30—C6	1.420 (7)
Mo4—O16	1.914 (4)	N1—C2	1.491 (7)
Mo4—O23	2.265 (4)	N2—C5	1.523 (7)
Mo4—O24	2.275 (4)	C1—C2	1.548 (8)
Mo5—O9	1.704 (4)	C2—C3	1.523 (8)
Mo5—O10	1.713 (4)	C4—C5	1.529 (7)
Mo5—O16	1.929 (4)	C5—C6	1.530 (8)
Mo5—O17	1.931 (4)	O27—H4	0.840
Mo5—O19	2.288 (4)	O30—H11	0.840
Mo5—O24	2.292 (4)	N1—H5	0.910
Mo6—O11	1.714 (4)	N1—H6	0.910
Mo6—O12	1.719 (4)	N1—H7	0.910
Mo6—O17	1.934 (4)	N2—H13	0.910
Mo6—O18	1.946 (4)	N2—H12	0.910
Mo6—O19	2.274 (4)	N2—H14	0.910
Mo6—O20	2.262 (3)	C2—H1	1.000
Rb1—O3	3.138 (4)	C3—H2	0.990
Rb1—O8^i^	2.858 (4)	C3—H3	0.990
Rb1—O11^ii^	2.855 (4)	C5—H8	1.000
Rb1—O12^ii^	3.099 (4)	C6—H9	0.990
Rb1—O14	2.737 (4)	C6—H10	0.990
			
Co1···O13	3.390 (4)	O7···H12^v^	3.2919
Co1···O14	3.387 (4)	O7···H14^v^	3.0848
Co1···O15	3.380 (3)	O8···H13^v^	2.7395
Co1···O16	3.364 (4)	O8···H12^v^	2.3132
Co1···O17	3.374 (4)	O8···H14^v^	3.3485
Co1···O18	3.389 (3)	O10···H8^iii^	2.4384
Co2···O29	3.349 (4)	O11···H12^iii^	2.3729
O1···O12	3.252 (6)	O11···H14^iii^	3.4299
O1···O19^iii^	2.714 (5)	O11···H8^iii^	2.6886
O1···O31^iii^	3.344 (6)	O13···H13^vi^	3.3249
O2···O3	3.305 (5)	O13···H12^vi^	3.2702
O2···O6^iv^	3.528 (5)	O13···H14^vi^	1.9329
O4···O6	3.287 (5)	O13···H8^vi^	3.4279
O4···O7^v^	3.494 (6)	O13···H10^vi^	2.7195
O4···O30^vi^	3.122 (6)	O25···H1	2.5619
O4···N2^vi^	3.110 (6)	O25···H2	2.7950
O4···C6^vi^	3.427 (7)	O26···H1	3.0852
O5···O7	3.214 (6)	O26···H4	3.3433
O5···O24^i^	2.710 (5)	O26···H5	3.5492
O5···O31	3.414 (5)	O26···H6	2.4944
O7···O22^i^	2.647 (5)	O26···H7	2.6156
O7···N2^v^	3.387 (6)	O27···H1	3.2887
O8···O9	3.194 (6)	O27···H5	3.2180
O8···N2^v^	2.912 (6)	O27···H6	2.6237
O9···O32^v^	2.789 (6)	O28···H13	2.6015
O10···O11	3.202 (5)	O28···H12	2.6064
O10···C5^iii^	3.383 (7)	O28···H14	3.5942
O11···N2^iii^	3.073 (6)	O28···H8	2.9795
O11···C4^iii^	3.476 (6)	O29···H8	2.6561
O11···C5^iii^	3.184 (7)	O29···H9	2.5982
O12···O21^iii^	2.790 (5)	O30···H13	2.7231
O13···N2^vi^	2.827 (6)	O30···H14	3.2090
O13···C5^vi^	3.453 (6)	O30···H8	3.2665
O13···C6^vi^	3.472 (7)	O36···H9^ix^	3.0878
O15···O31^v^	2.731 (5)	N1···H2	3.3264
O18···O31^iii^	2.915 (5)	N1···H3	2.6702
O23···O31	2.637 (5)	N1···H4	2.6186
O25···C3	3.036 (7)	N2···H9	3.3613
O26···O27	3.323 (6)	N2···H10	2.6694
O26···N1	2.647 (6)	C1···H2	2.7114
O26···C3	3.342 (7)	C1···H3	3.3743
O27···N1	2.940 (6)	C1···H4	3.2060
O27···C1	2.950 (7)	C1···H5	3.2783
O28···N2	2.691 (6)	C1···H6	2.6629
O28···C6	3.528 (7)	C1···H7	2.6307
O29···O30	3.215 (6)	C2···H4	2.5901
O29···O33^vii^	3.469 (6)	C3···H5	2.6429
O29···O34	2.592 (6)	C3···H6	2.6418
O29···C6	2.826 (7)	C3···H7	3.2631
O30···N2	2.978 (6)	C4···H13	2.6571
O30···C4	2.893 (7)	C4···H12	2.7012
O33···C4	3.593 (6)	C4···H14	3.2938
O34···C4	3.130 (6)	C4···H9	2.7369
O1···O38^v^	3.283 (6)	C4···H10	3.3691
O2···O38^iv^	3.594 (7)	C5···H11	3.1558
O2···N1	2.944 (6)	C6···H13	2.7233
O2···C2	3.283 (6)	C6···H12	3.2912
O2···C3	3.190 (7)	C6···H14	2.6329
O3···O42^viii^	3.236 (6)	H1···H2	2.3511
O3···C2	3.152 (7)	H1···H3	2.3608
O3···C3	3.081 (7)	H1···H4	3.4866
O4···O39^ix^	3.030 (6)	H1···H5	2.2942
O4···O42^viii^	3.216 (6)	H1···H6	2.7796
O4···O43^vi^	3.154 (6)	H1···H7	2.3045
O5···O38	3.198 (7)	H2···H4	2.6673
O6···O39^ix^	3.009 (6)	H2···H5	3.5292
O6···O41^x^	2.916 (6)	H2···H6	3.5481
O6···N1^x^	3.101 (6)	H13···H8	2.7983
O7···O38	3.091 (7)	H13···H10	2.9751
O9···O26^iii^	3.050 (6)	H13···H11	3.4765
O10···O37^xi^	2.721 (6)	H12···H8	2.2966
O10···O41^ii^	3.455 (6)	H12···H10	3.4808
O13···O38^v^	3.541 (7)	H14···H8	2.3477
O14···O39^ix^	3.339 (6)	H14···H9	3.5184
O15···O38	2.869 (5)	H14···H10	2.4393
O16···O41^ii^	2.894 (6)	H3···H4	2.2173
O16···O43^v^	3.520 (6)	H3···H5	2.4834
O17···N1^iii^	2.905 (6)	H3···H6	2.9242
O17···C2^iii^	3.403 (6)	H3···H7	3.4992
O18···O38^iv^	2.746 (5)	H4···H5	2.8423
O20···O38^v^	2.566 (5)	H4···H6	2.1224
O25···O35	2.749 (6)	H4···H7	3.4521
O25···O45^viii^	2.837 (7)	H8···H9	2.3377
O26···O9^ii^	3.050 (6)	H8···H10	2.3876
O26···O32^iv^	3.411 (6)	H9···H11	2.2061
O26···O39^vii^	3.313 (6)	H10···H11	2.2058
O26···O40^vii^	2.713 (6)	Mo1···H3	3.5841
O26···O44^iv^	2.729 (6)	Mo5···H7^iii^	3.3029
O27···O34^viii^	3.562 (6)	O2···H1	3.1673
O27···O36^viii^	2.766 (6)	O2···H3	2.3869
O27···O39^vii^	3.337 (6)	O2···H5	2.1144
O27···O41	2.859 (6)	O2···H6	3.4440
O27···O44^iv^	3.511 (6)	O2···H7	3.4959
O27···O45^iv^	3.347 (6)	O3···H1	2.4073
O28···O43	3.367 (6)	O3···H2	2.8346
O29···O37^vii^	2.758 (6)	O3···H3	2.8059
O29···O40	3.386 (6)	O5···H5^x^	3.5281
O30···O40	2.708 (6)	O5···H7^x^	3.2581
O30···O42^vii^	2.928 (6)	O6···H5^x^	2.6821
O30···O43	2.931 (7)	O6···H6^x^	2.7716
O32···O26^x^	3.411 (6)	O6···H7^x^	3.4202
O32···O39^ix^	2.793 (6)	O9···H7^iii^	2.9001
O33···O37	3.159 (6)	O12···H1^iii^	3.1126
O33···O40^ix^	2.762 (6)	O13···H3	3.4190
O33···O43	2.864 (6)	O17···H1^iii^	3.1189
O34···O27^xii^	3.562 (6)	O17···H5^iii^	3.1826
O34···O37	2.693 (6)	O17···H6^iii^	3.5719
O34···O45^viii^	2.812 (6)	O17···H7^iii^	2.0891
O35···O25	2.749 (6)	O27···H9^vi^	3.4869
O35···O42^viii^	2.672 (6)	O35···H1	3.3071
O36···O27^xii^	2.766 (6)	O35···H2	3.5739
O36···O42^viii^	3.467 (6)	O36···H2^xii^	2.9821
O36···O44	2.711 (6)	O36···H4^xii^	3.4952
O36···C3^xii^	3.401 (7)	O37···H8^ix^	3.3473
O37···O10^xiii^	2.721 (6)	O37···H9^ix^	2.9248
O37···O29^ix^	2.758 (6)	O38···H12^v^	3.4037
O37···O33	3.159 (6)	O38···H14^v^	3.4937
O37···O34	2.693 (6)	O39···H4^ix^	2.6354
O37···O40	2.770 (6)	O39···H5^ix^	3.4609
O38···O1^i^	3.283 (6)	O39···H6^ix^	2.1396
O38···O2^x^	3.594 (7)	O39···H7^ix^	3.3673
O38···O5	3.198 (7)	O40···H9	3.2207
O38···O7	3.091 (7)	O40···H11	2.5578
O38···O13^i^	3.541 (7)	O41···H3	3.2008
O38···O15	2.869 (5)	O41···H4	2.2367
O38···O18^x^	2.746 (5)	O41···H5	3.5017
O38···O20^i^	2.566 (5)	O41···H6	3.3191
O39···O4^vii^	3.030 (6)	O42···H2^xii^	3.3487
O39···O6^vii^	3.009 (6)	O42···H10^ix^	3.5090
O39···O14^vii^	3.339 (6)	O42···H11^ix^	2.2422
O39···O26^ix^	3.313 (6)	O43···H13	2.1120
O39···O27^ix^	3.337 (6)	O43···H12	3.4863
O39···O32^vii^	2.793 (6)	O43···H14	3.4463
O39···O41^ix^	3.328 (6)	O43···H11	3.4813
O39···O42^xiv^	3.197 (6)	O44···H2^xii^	3.1971
O39···O44^vii^	3.147 (6)	O44···H4^x^	3.4995
O39···O45^vii^	2.909 (6)	O44···H11^ix^	3.0896
O39···N1^ix^	3.041 (6)	O45···H4^x^	3.0294
O40···O26^ix^	2.713 (6)	O45···H11^vi^	3.5431
O40···O29	3.386 (6)	C3···H9^vi^	3.3681
O40···O30	2.708 (6)	C3···H10^vi^	2.9592
O40···O33^vii^	2.762 (6)	C6···H2^xvii^	3.1970
O40···O37	2.770 (6)	C6···H3^xvii^	3.2224
O40···O44^vii^	3.491 (6)	H1···O2	3.1673
O40···C1^ix^	3.536 (7)	H1···O3	2.4073
O40···C6	3.500 (7)	H1···O12^ii^	3.1126
O41···O6^iv^	2.916 (6)	H1···O17^ii^	3.1189
O41···O10^iii^	3.455 (6)	H1···O35	3.3071
O41···O16^iii^	2.894 (6)	H2···O3	2.8346
O41···O27	2.859 (6)	H2···O35	3.5739
O41···O39^vii^	3.328 (6)	H2···O36^viii^	2.9821
O41···O43^xv^	3.046 (7)	H2···O42^viii^	3.3487
O41···C3	3.539 (8)	H2···O44^viii^	3.1971
O42···O3^xii^	3.236 (6)	H2···C6^vi^	3.1970
O42···O4^xii^	3.216 (6)	H2···H9^vi^	2.9953
O42···O30^ix^	2.928 (6)	H2···H10^vi^	2.6152
O42···O35^xii^	2.672 (6)	H2···H11^vi^	3.2630
O42···O36^xii^	3.467 (6)	H13···O43	2.1120
O42···O39^xvi^	3.197 (6)	H12···O38^i^	3.4037
O42···O44	2.768 (7)	H12···O43	3.4863
O42···O45^xii^	3.092 (7)	H14···O38^i^	3.4937
O43···O4^xvii^	3.154 (6)	H14···O43	3.4463
O43···O16^i^	3.520 (6)	H3···Mo1	3.5841
O43···O28	3.367 (6)	H3···O2	2.3869
O43···O30	2.931 (7)	H3···O3	2.8059
O43···O33	2.864 (6)	H3···O13	3.4190
O43···O41^xviii^	3.046 (7)	H3···O41	3.2008
O43···O45^xvii^	3.294 (7)	H3···C6^vi^	3.2224
O43···N2	3.013 (7)	H3···H9^vi^	3.1293
O44···O26^x^	2.729 (6)	H3···H10^vi^	2.4462
O44···O27^x^	3.511 (6)	H4···O36^viii^	3.4952
O44···O36	2.711 (6)	H4···O39^vii^	2.6354
O44···O39^ix^	3.147 (6)	H4···O41	2.2367
O44···O40^ix^	3.491 (6)	H4···O44^iv^	3.4995
O44···O42	2.768 (7)	H4···O45^iv^	3.0294
O44···O45	2.805 (7)	H5···O2	2.1144
O44···C1^x^	3.494 (7)	H5···O5^iv^	3.5281
O45···O25^xii^	2.837 (7)	H5···O6^iv^	2.6821
O45···O27^x^	3.347 (6)	H5···O17^ii^	3.1826
O45···O34^xii^	2.812 (6)	H5···O39^vii^	3.4609
O45···O39^ix^	2.909 (6)	H5···O41	3.5017
O45···O42^viii^	3.092 (7)	H6···O2	3.4440
O45···O43^vi^	3.294 (7)	H6···O6^iv^	2.7716
O45···O44	2.805 (7)	H6···O17^ii^	3.5719
N1···O2	2.944 (6)	H6···O39^vii^	2.1396
N1···O6^iv^	3.101 (6)	H6···O41	3.3191
N1···O17^ii^	2.905 (6)	H7···Mo5^ii^	3.3029
N1···O39^vii^	3.041 (6)	H7···O2	3.4959
N2···O43	3.013 (7)	H7···O5^iv^	3.2581
C1···O40^vii^	3.536 (7)	H7···O6^iv^	3.4202
C1···O44^iv^	3.494 (7)	H7···O9^ii^	2.9001
C2···O2	3.283 (6)	H7···O17^ii^	2.0891
C2···O3	3.152 (7)	H7···O39^vii^	3.3673
C2···O17^ii^	3.403 (6)	H8···O37^vii^	3.3473
C3···O2	3.190 (7)	H9···O27^xvii^	3.4869
C3···O3	3.081 (7)	H9···O37^vii^	2.9248
C3···O36^viii^	3.401 (7)	H9···O40	3.2207
C3···O41	3.539 (8)	H9···C3^xvii^	3.3681
C6···O40	3.500 (7)	H9···H2^xvii^	2.9953
Mo2···H14^vi^	3.0638	H9···H3^xvii^	3.1293
Mo2···H10^vi^	3.2343	H10···O42^vii^	3.5090
Mo4···H13^v^	3.5913	H10···C3^xvii^	2.9592
Mo4···H12^v^	3.4589	H10···H2^xvii^	2.6152
Rb1···H12	3.3372	H10···H3^xvii^	2.4462
O3···H10^vi^	3.0200	H11···O40	2.5578
O4···H13^vi^	2.8030	H11···O42^vii^	2.2422
O4···H14^vi^	2.6260	H11···O43	3.4813
O4···H10^vi^	2.9750	H11···O44^vii^	3.0896
O4···H11^vi^	3.2797	H11···O45^xvii^	3.5431
O7···H13^v^	3.2320	H11···H2^xvii^	3.2630
			
O1—Mo1—O2	106.26 (19)	O12^ii^—Rb1—O28	121.37 (10)
O1—Mo1—O13	98.55 (16)	O12^ii^—Rb1—O31	59.59 (10)
O1—Mo1—O18	99.41 (16)	O12^ii^—Rb1—O32	160.80 (11)
O1—Mo1—O20	94.66 (15)	O12^ii^—Rb1—O35	108.16 (10)
O1—Mo1—O21	161.17 (15)	O14—Rb1—O28	149.48 (10)
O2—Mo1—O13	101.91 (16)	O14—Rb1—O31	71.23 (10)
O2—Mo1—O18	95.28 (16)	O14—Rb1—O32	85.65 (11)
O2—Mo1—O20	157.08 (15)	O14—Rb1—O35	101.05 (11)
O2—Mo1—O21	91.95 (15)	O28—Rb1—O31	134.06 (9)
O13—Mo1—O18	150.50 (14)	O28—Rb1—O32	64.74 (10)
O13—Mo1—O20	83.71 (13)	O28—Rb1—O35	57.77 (10)
O13—Mo1—O21	72.44 (13)	O31—Rb1—O32	131.53 (10)
O18—Mo1—O20	71.68 (12)	O31—Rb1—O35	165.42 (10)
O18—Mo1—O21	83.28 (12)	O32—Rb1—O35	57.89 (11)
O20—Mo1—O21	68.32 (11)	O19—Co1—O20	84.13 (14)
O3—Mo2—O4	106.40 (17)	O19—Co1—O21	95.27 (14)
O3—Mo2—O13	101.74 (16)	O19—Co1—O22	179.37 (14)
O3—Mo2—O14	96.02 (16)	O19—Co1—O23	96.87 (14)
O3—Mo2—O21	91.44 (14)	O19—Co1—O24	84.19 (14)
O3—Mo2—O22	157.18 (15)	O20—Co1—O21	84.43 (13)
O4—Mo2—O13	96.50 (16)	O20—Co1—O22	95.24 (13)
O4—Mo2—O14	100.17 (16)	O20—Co1—O23	178.98 (14)
O4—Mo2—O21	160.79 (15)	O20—Co1—O24	95.97 (14)
O4—Mo2—O22	94.94 (15)	O21—Co1—O22	84.74 (14)
O13—Mo2—O14	151.07 (14)	O21—Co1—O23	95.64 (14)
O13—Mo2—O21	72.40 (12)	O21—Co1—O24	179.29 (14)
O13—Mo2—O22	83.38 (13)	O22—Co1—O23	83.75 (14)
O14—Mo2—O21	84.61 (13)	O22—Co1—O24	95.80 (14)
O14—Mo2—O22	71.82 (13)	O23—Co1—O24	83.96 (14)
O21—Mo2—O22	68.69 (11)	O28—Co2—O32	95.09 (14)
O5—Mo3—O6	106.47 (18)	O28—Co2—O33	83.78 (14)
O5—Mo3—O14	97.22 (16)	O28—Co2—O34	91.63 (14)
O5—Mo3—O15	100.19 (16)	O28—Co2—O35	87.62 (14)
O5—Mo3—O22	159.57 (15)	O28—Co2—O36	171.73 (16)
O5—Mo3—O23	94.01 (15)	O32—Co2—O33	87.67 (15)
O6—Mo3—O14	100.99 (16)	O32—Co2—O34	172.49 (15)
O6—Mo3—O15	97.04 (16)	O32—Co2—O35	85.04 (15)
O6—Mo3—O22	92.83 (15)	O32—Co2—O36	90.17 (16)
O6—Mo3—O23	158.31 (15)	O33—Co2—O34	96.41 (15)
O14—Mo3—O15	150.19 (14)	O33—Co2—O35	168.19 (16)
O14—Mo3—O22	72.02 (13)	O33—Co2—O36	90.08 (16)
O14—Mo3—O23	82.99 (13)	O34—Co2—O35	91.89 (15)
O15—Mo3—O22	83.62 (12)	O34—Co2—O36	83.53 (15)
O15—Mo3—O23	71.85 (13)	O35—Co2—O36	99.20 (17)
O22—Mo3—O23	67.91 (11)	Mo2—O3—Rb1	100.10 (15)
Mo5—Mo4—O7	130.93 (13)	Mo4—O8—Rb1^v^	154.38 (19)
Mo5—Mo4—O8	88.76 (12)	Mo6—O11—Rb1^iii^	104.41 (16)
Mo5—Mo4—O15	126.59 (10)	Mo6—O12—Rb1^iii^	95.20 (15)
Mo5—Mo4—O16	31.70 (11)	Mo1—O13—Mo2	118.07 (17)
Mo5—Mo4—O23	85.56 (8)	Mo2—O14—Mo3	118.38 (18)
Mo5—Mo4—O24	44.39 (8)	Mo2—O14—Rb1	108.75 (14)
O7—Mo4—O8	106.22 (18)	Mo3—O14—Rb1	132.38 (16)
O7—Mo4—O15	98.85 (16)	Mo3—O15—Mo4	117.05 (16)
O7—Mo4—O16	99.30 (17)	Mo4—O16—Mo5	116.88 (18)
O7—Mo4—O23	93.16 (15)	Mo5—O17—Mo6	116.42 (18)
O7—Mo4—O24	160.57 (16)	Mo1—O18—Mo6	117.03 (16)
O8—Mo4—O15	95.26 (16)	Mo5—O19—Mo6	92.14 (12)
O8—Mo4—O16	101.74 (16)	Mo5—O19—Co1	103.93 (15)
O8—Mo4—O23	158.36 (15)	Mo6—O19—Co1	103.34 (14)
O8—Mo4—O24	92.94 (15)	Mo1—O20—Mo6	94.15 (11)
O15—Mo4—O16	150.49 (14)	Mo1—O20—Co1	104.13 (14)
O15—Mo4—O23	71.86 (13)	Mo6—O20—Co1	103.67 (14)
O15—Mo4—O24	82.20 (12)	Mo1—O21—Mo2	92.51 (12)
O16—Mo4—O23	84.08 (14)	Mo1—O21—Co1	103.11 (14)
O16—Mo4—O24	73.07 (14)	Mo2—O21—Co1	102.89 (14)
O23—Mo4—O24	68.58 (11)	Mo2—O22—Mo3	92.91 (12)
Mo4—Mo5—O9	88.49 (13)	Mo2—O22—Co1	103.68 (14)
Mo4—Mo5—O10	127.50 (14)	Mo3—O22—Co1	104.17 (14)
Mo4—Mo5—O16	31.42 (11)	Mo3—O23—Mo4	93.89 (11)
Mo4—Mo5—O17	125.79 (11)	Mo3—O23—Co1	104.17 (14)
Mo4—Mo5—O19	84.06 (9)	Mo4—O23—Co1	103.81 (15)
Mo4—Mo5—O24	43.98 (8)	Mo4—O24—Mo5	91.63 (12)
O9—Mo5—O10	106.67 (18)	Mo4—O24—Co1	103.59 (14)
O9—Mo5—O16	100.31 (17)	Mo5—O24—Co1	103.84 (15)
O9—Mo5—O17	97.62 (17)	Rb1—O28—Co2	89.21 (11)
O9—Mo5—O19	160.55 (16)	Rb1—O28—C4	128.6 (3)
O9—Mo5—O24	94.26 (16)	Co2—O28—C4	126.0 (3)
O10—Mo5—O16	96.11 (17)	Rb1—O32—Co2	91.66 (12)
O10—Mo5—O17	102.17 (17)	Rb1—O35—Co2	89.61 (13)
O10—Mo5—O19	92.05 (15)	O25—C1—O26	127.5 (5)
O10—Mo5—O24	157.81 (15)	O25—C1—C2	116.4 (5)
O16—Mo5—O17	149.46 (15)	O26—C1—C2	116.1 (5)
O16—Mo5—O19	82.45 (14)	N1—C2—C1	110.0 (4)
O16—Mo5—O24	72.42 (13)	N1—C2—C3	110.7 (4)
O17—Mo5—O19	72.73 (13)	C1—C2—C3	110.3 (5)
O17—Mo5—O24	81.81 (13)	O27—C3—C2	112.1 (5)
O19—Mo5—O24	68.03 (12)	O28—C4—O29	126.5 (5)
O11—Mo6—O12	105.70 (18)	O28—C4—C5	118.2 (4)
O11—Mo6—O17	101.14 (16)	O29—C4—C5	115.3 (5)
O11—Mo6—O18	95.36 (15)	N2—C5—C4	110.9 (4)
O11—Mo6—O19	92.39 (15)	N2—C5—C6	110.8 (4)
O11—Mo6—O20	158.22 (15)	C4—C5—C6	110.7 (5)
O12—Mo6—O17	98.83 (16)	O30—C6—C5	110.6 (5)
O12—Mo6—O18	99.02 (16)	C3—O27—H4	109.473
O12—Mo6—O19	161.42 (15)	C6—O30—H11	109.466
O12—Mo6—O20	94.11 (15)	C2—N1—H5	109.471
O17—Mo6—O18	151.34 (14)	C2—N1—H6	109.455
O17—Mo6—O19	73.00 (13)	C2—N1—H7	109.469
O17—Mo6—O20	84.30 (13)	H5—N1—H6	109.477
O18—Mo6—O19	83.13 (13)	H5—N1—H7	109.484
O18—Mo6—O20	72.22 (13)	H6—N1—H7	109.471
O19—Mo6—O20	68.83 (11)	C5—N2—H13	109.480
O3—Rb1—O8^i^	167.01 (10)	C5—N2—H12	109.476
O3—Rb1—O11^ii^	109.60 (10)	C5—N2—H14	109.470
O3—Rb1—O12^ii^	65.85 (10)	H13—N2—H12	109.468
O3—Rb1—O14	54.60 (10)	H13—N2—H14	109.467
O3—Rb1—O28	117.88 (9)	H12—N2—H14	109.467
O3—Rb1—O31	104.15 (9)	N1—C2—H1	108.575
O3—Rb1—O32	95.04 (10)	C1—C2—H1	108.597
O3—Rb1—O35	61.86 (10)	C3—C2—H1	108.589
O8^i^—Rb1—O11^ii^	79.66 (10)	O27—C3—H2	109.176
O8^i^—Rb1—O12^ii^	115.90 (10)	O27—C3—H3	109.174
O8^i^—Rb1—O14	112.41 (11)	C2—C3—H2	109.171
O8^i^—Rb1—O28	72.99 (10)	C2—C3—H3	109.182
O8^i^—Rb1—O31	68.50 (10)	H2—C3—H3	107.896
O8^i^—Rb1—O32	83.11 (11)	N2—C5—H8	108.109
O8^i^—Rb1—O35	126.03 (11)	C4—C5—H8	108.111
O11^ii^—Rb1—O12^ii^	54.54 (10)	C6—C5—H8	108.105
O11^ii^—Rb1—O14	136.93 (11)	O30—C6—H9	109.516
O11^ii^—Rb1—O28	73.03 (10)	O30—C6—H10	109.526
O11^ii^—Rb1—O31	76.24 (10)	C5—C6—H9	109.517
O11^ii^—Rb1—O32	137.42 (11)	C5—C6—H10	109.524
O11^ii^—Rb1—O35	103.69 (10)	H9—C6—H10	108.087
O12^ii^—Rb1—O14	84.44 (10)		
			
O1—Mo1—O13—Mo2	177.9 (2)	O17—Mo5—O19—Co1	−88.44 (16)
O1—Mo1—O18—Mo6	72.4 (2)	O19—Mo5—O17—Mo6	−21.03 (16)
O1—Mo1—O20—Mo6	−83.55 (16)	O17—Mo5—O24—Mo4	179.52 (14)
O1—Mo1—O20—Co1	171.23 (17)	O17—Mo5—O24—Co1	75.09 (16)
O2—Mo1—O13—Mo2	69.2 (2)	O24—Mo5—O17—Mo6	−90.48 (19)
O2—Mo1—O18—Mo6	179.83 (19)	O19—Mo5—O24—Mo4	104.90 (13)
O2—Mo1—O20—Mo6	72.5 (4)	O19—Mo5—O24—Co1	0.47 (12)
O2—Mo1—O20—Co1	−32.7 (5)	O24—Mo5—O19—Mo6	103.84 (13)
O2—Mo1—O21—Mo2	−87.70 (15)	O24—Mo5—O19—Co1	−0.47 (12)
O2—Mo1—O21—Co1	168.45 (17)	O11—Mo6—O12—Rb1^iii^	3.34 (17)
O13—Mo1—O18—Mo6	−54.4 (4)	O12—Mo6—O11—Rb1^iii^	−3.73 (19)
O18—Mo1—O13—Mo2	−55.1 (4)	O11—Mo6—O17—Mo5	−68.0 (2)
O13—Mo1—O20—Mo6	178.34 (13)	O17—Mo6—O11—Rb1^iii^	−106.31 (15)
O13—Mo1—O20—Co1	73.12 (15)	O11—Mo6—O18—Mo1	−178.75 (19)
O20—Mo1—O13—Mo2	−88.30 (18)	O18—Mo6—O11—Rb1^iii^	97.23 (14)
O13—Mo1—O21—Mo2	14.17 (12)	O11—Mo6—O19—Mo5	85.10 (15)
O13—Mo1—O21—Co1	−89.68 (15)	O11—Mo6—O19—Co1	−170.05 (17)
O21—Mo1—O13—Mo2	−19.09 (15)	O19—Mo6—O11—Rb1^iii^	−179.45 (13)
O18—Mo1—O20—Mo6	14.84 (12)	O11—Mo6—O20—Mo1	−72.3 (4)
O18—Mo1—O20—Co1	−90.39 (15)	O11—Mo6—O20—Co1	33.3 (5)
O20—Mo1—O18—Mo6	−19.47 (15)	O20—Mo6—O11—Rb1^iii^	151.0 (3)
O18—Mo1—O21—Mo2	177.21 (13)	O12—Mo6—O17—Mo5	−176.0 (2)
O18—Mo1—O21—Co1	73.36 (15)	O17—Mo6—O12—Rb1^iii^	107.63 (13)
O21—Mo1—O18—Mo6	−88.82 (17)	O12—Mo6—O18—Mo1	−71.9 (2)
O20—Mo1—O21—Mo2	104.29 (13)	O18—Mo6—O12—Rb1^iii^	−94.88 (13)
O20—Mo1—O21—Co1	0.44 (12)	O12—Mo6—O20—Mo1	83.31 (15)
O21—Mo1—O20—Mo6	104.78 (13)	O12—Mo6—O20—Co1	−171.05 (17)
O21—Mo1—O20—Co1	−0.45 (12)	O20—Mo6—O12—Rb1^iii^	−167.51 (11)
O4—Mo2—O3—Rb1	−108.37 (16)	O17—Mo6—O18—Mo1	56.0 (4)
O3—Mo2—O13—Mo1	−68.6 (2)	O18—Mo6—O17—Mo5	56.0 (4)
O13—Mo2—O3—Rb1	151.16 (12)	O17—Mo6—O19—Mo5	−15.82 (13)
O3—Mo2—O14—Mo3	180.0 (2)	O17—Mo6—O19—Co1	89.03 (16)
O3—Mo2—O14—Rb1	7.03 (17)	O19—Mo6—O17—Mo5	21.13 (16)
O14—Mo2—O3—Rb1	−5.89 (15)	O17—Mo6—O20—Mo1	−178.21 (14)
O3—Mo2—O21—Mo1	87.80 (15)	O17—Mo6—O20—Co1	−72.57 (15)
O3—Mo2—O21—Co1	−168.14 (17)	O20—Mo6—O17—Mo5	90.70 (18)
O21—Mo2—O3—Rb1	78.84 (12)	O18—Mo6—O19—Mo5	−179.78 (14)
O3—Mo2—O22—Mo3	−74.7 (4)	O18—Mo6—O19—Co1	−74.93 (15)
O3—Mo2—O22—Co1	30.6 (5)	O19—Mo6—O18—Mo1	89.48 (17)
O22—Mo2—O3—Rb1	50.2 (4)	O18—Mo6—O20—Mo1	−14.86 (12)
O4—Mo2—O13—Mo1	−176.89 (19)	O18—Mo6—O20—Co1	90.78 (15)
O4—Mo2—O14—Mo3	−72.1 (3)	O20—Mo6—O18—Mo1	19.57 (15)
O4—Mo2—O14—Rb1	114.93 (16)	O19—Mo6—O20—Mo1	−104.26 (13)
O4—Mo2—O22—Mo3	84.66 (15)	O19—Mo6—O20—Co1	1.38 (12)
O4—Mo2—O22—Co1	−170.00 (17)	O20—Mo6—O19—Mo5	−106.23 (13)
O13—Mo2—O14—Mo3	52.1 (4)	O20—Mo6—O19—Co1	−1.38 (12)
O13—Mo2—O14—Rb1	−120.8 (3)	O3—Rb1—O11^ii^—Mo6^ii^	40.54 (18)
O14—Mo2—O13—Mo1	58.1 (4)	O11^ii^—Rb1—O3—Mo2	−129.20 (14)
O13—Mo2—O21—Mo1	−14.06 (12)	O3—Rb1—O12^ii^—Mo6^ii^	−142.79 (18)
O13—Mo2—O21—Co1	90.00 (15)	O12^ii^—Rb1—O3—Mo2	−95.77 (16)
O21—Mo2—O13—Mo1	19.17 (15)	O3—Rb1—O14—Mo2	−4.67 (12)
O13—Mo2—O22—Mo3	−179.35 (13)	O3—Rb1—O14—Mo3	−176.3 (3)
O13—Mo2—O22—Co1	−74.01 (15)	O14—Rb1—O3—Mo2	5.09 (13)
O22—Mo2—O13—Mo1	88.87 (18)	O3—Rb1—O28—Co2	−53.19 (14)
O14—Mo2—O21—Mo1	−176.28 (14)	O3—Rb1—O28—C4	84.8 (3)
O14—Mo2—O21—Co1	−72.22 (15)	O28—Rb1—O3—Mo2	150.14 (13)
O21—Mo2—O14—Mo3	89.09 (19)	O31—Rb1—O3—Mo2	−49.02 (16)
O21—Mo2—O14—Rb1	−83.85 (14)	O3—Rb1—O32—Co2	90.18 (13)
O14—Mo2—O22—Mo3	−14.44 (12)	O32—Rb1—O3—Mo2	86.11 (15)
O14—Mo2—O22—Co1	90.90 (16)	O3—Rb1—O35—Co2	−156.67 (16)
O22—Mo2—O14—Mo3	19.78 (17)	O35—Rb1—O3—Mo2	135.39 (18)
O22—Mo2—O14—Rb1	−153.17 (17)	O8^i^—Rb1—O11^ii^—Mo6^ii^	−130.02 (17)
O21—Mo2—O22—Mo3	−105.70 (13)	O11^ii^—Rb1—O8^i^—Mo4^i^	105.3 (5)
O21—Mo2—O22—Co1	−0.35 (12)	O8^i^—Rb1—O12^ii^—Mo6^ii^	51.42 (18)
O22—Mo2—O21—Mo1	−103.71 (13)	O12^ii^—Rb1—O8^i^—Mo4^i^	63.4 (5)
O22—Mo2—O21—Co1	0.35 (12)	O8^i^—Rb1—O14—Mo2	175.29 (13)
O5—Mo3—O14—Mo2	178.2 (2)	O8^i^—Rb1—O14—Mo3	3.7 (3)
O5—Mo3—O14—Rb1	−10.9 (3)	O14—Rb1—O8^i^—Mo4^i^	−31.4 (5)
O5—Mo3—O15—Mo4	70.6 (2)	O8^i^—Rb1—O28—Co2	119.11 (13)
O5—Mo3—O22—Mo2	75.1 (5)	O8^i^—Rb1—O28—C4	−102.9 (3)
O5—Mo3—O22—Co1	−29.8 (5)	O28—Rb1—O8^i^—Mo4^i^	−179.4 (5)
O5—Mo3—O23—Mo4	−83.85 (15)	O31—Rb1—O8^i^—Mo4^i^	26.2 (4)
O5—Mo3—O23—Co1	170.86 (17)	O8^i^—Rb1—O32—Co2	−102.75 (13)
O6—Mo3—O14—Mo2	69.7 (3)	O32—Rb1—O8^i^—Mo4^i^	−113.8 (5)
O6—Mo3—O14—Rb1	−119.3 (3)	O8^i^—Rb1—O35—Co2	11.12 (18)
O6—Mo3—O15—Mo4	178.83 (19)	O35—Rb1—O8^i^—Mo4^i^	−155.2 (4)
O6—Mo3—O22—Mo2	−86.02 (15)	O11^ii^—Rb1—O12^ii^—Mo6^ii^	−2.37 (12)
O6—Mo3—O22—Co1	169.08 (17)	O12^ii^—Rb1—O11^ii^—Mo6^ii^	2.44 (13)
O6—Mo3—O23—Mo4	77.2 (4)	O11^ii^—Rb1—O14—Mo2	76.3 (2)
O6—Mo3—O23—Co1	−28.1 (5)	O11^ii^—Rb1—O14—Mo3	−95.3 (3)
O14—Mo3—O15—Mo4	−54.2 (4)	O14—Rb1—O11^ii^—Mo6^ii^	−18.2 (3)
O15—Mo3—O14—Mo2	−56.4 (4)	O11^ii^—Rb1—O28—Co2	−156.80 (13)
O15—Mo3—O14—Rb1	114.5 (3)	O11^ii^—Rb1—O28—C4	−18.9 (3)
O14—Mo3—O22—Mo2	14.62 (13)	O28—Rb1—O11^ii^—Mo6^ii^	154.77 (17)
O14—Mo3—O22—Co1	−90.28 (16)	O31—Rb1—O11^ii^—Mo6^ii^	−59.83 (15)
O22—Mo3—O14—Mo2	−19.67 (17)	O11^ii^—Rb1—O32—Co2	−36.2 (2)
O22—Mo3—O14—Rb1	151.3 (3)	O32—Rb1—O11^ii^—Mo6^ii^	162.24 (13)
O14—Mo3—O23—Mo4	179.36 (14)	O11^ii^—Rb1—O35—Co2	98.18 (13)
O14—Mo3—O23—Co1	74.06 (16)	O35—Rb1—O11^ii^—Mo6^ii^	105.17 (16)
O23—Mo3—O14—Mo2	−88.66 (19)	O12^ii^—Rb1—O14—Mo2	59.53 (15)
O23—Mo3—O14—Rb1	82.3 (2)	O12^ii^—Rb1—O14—Mo3	−112.1 (3)
O15—Mo3—O22—Mo2	177.20 (14)	O14—Rb1—O12^ii^—Mo6^ii^	163.66 (16)
O15—Mo3—O22—Co1	72.30 (15)	O12^ii^—Rb1—O28—Co2	−130.50 (11)
O22—Mo3—O15—Mo4	−89.09 (17)	O12^ii^—Rb1—O28—C4	7.4 (3)
O15—Mo3—O23—Mo4	15.57 (12)	O28—Rb1—O12^ii^—Mo6^ii^	−33.72 (19)
O15—Mo3—O23—Co1	−89.73 (16)	O31—Rb1—O12^ii^—Mo6^ii^	92.19 (16)
O23—Mo3—O15—Mo4	−20.29 (15)	O12^ii^—Rb1—O35—Co2	154.91 (11)
O22—Mo3—O23—Mo4	105.96 (13)	O35—Rb1—O12^ii^—Mo6^ii^	−96.50 (15)
O22—Mo3—O23—Co1	0.66 (12)	O14—Rb1—O28—Co2	13.7 (3)
O23—Mo3—O22—Mo2	104.23 (13)	O14—Rb1—O28—C4	151.6 (2)
O23—Mo3—O22—Co1	−0.67 (13)	O28—Rb1—O14—Mo2	−90.3 (3)
O7—Mo4—Mo5—O9	−108.32 (17)	O28—Rb1—O14—Mo3	98.1 (3)
O7—Mo4—Mo5—O10	1.64 (18)	O31—Rb1—O14—Mo2	119.27 (16)
O7—Mo4—Mo5—O16	4.43 (17)	O31—Rb1—O14—Mo3	−52.3 (2)
O7—Mo4—Mo5—O17	153.37 (17)	O14—Rb1—O32—Co2	144.03 (13)
O7—Mo4—Mo5—O19	89.66 (17)	O32—Rb1—O14—Mo2	−104.00 (16)
O7—Mo4—Mo5—O24	153.96 (18)	O32—Rb1—O14—Mo3	84.4 (3)
Mo5—Mo4—O8—Rb1^v^	94.4 (5)	O14—Rb1—O35—Co2	−117.37 (12)
O8—Mo4—Mo5—O9	2.23 (12)	O35—Rb1—O14—Mo2	−47.92 (16)
O8—Mo4—Mo5—O10	112.20 (12)	O35—Rb1—O14—Mo3	140.5 (2)
O8—Mo4—Mo5—O16	114.98 (12)	O31—Rb1—O28—Co2	153.09 (10)
O8—Mo4—Mo5—O17	−96.07 (12)	O31—Rb1—O28—C4	−69.0 (3)
O8—Mo4—Mo5—O19	−159.78 (12)	O28—Rb1—O32—Co2	−28.34 (11)
O8—Mo4—Mo5—O24	−95.48 (12)	O32—Rb1—O28—Co2	28.78 (11)
Mo5—Mo4—O15—Mo3	90.33 (17)	O32—Rb1—O28—C4	166.7 (3)
O15—Mo4—Mo5—O9	97.86 (12)	O28—Rb1—O35—Co2	38.75 (11)
O15—Mo4—Mo5—O10	−152.17 (12)	O35—Rb1—O28—Co2	−37.80 (11)
O15—Mo4—Mo5—O16	−149.39 (12)	O35—Rb1—O28—C4	100.1 (3)
O15—Mo4—Mo5—O17	−0.44 (12)	O31—Rb1—O32—Co2	−155.87 (10)
O15—Mo4—Mo5—O19	−64.15 (12)	O32—Rb1—O35—Co2	−39.72 (12)
O15—Mo4—Mo5—O24	0.15 (12)	O35—Rb1—O32—Co2	38.08 (11)
Mo5—Mo4—O16—Mo5	0.000 (13)	O19—Co1—O20—Mo1	96.40 (14)
O16—Mo4—Mo5—O9	−112.8 (2)	O19—Co1—O20—Mo6	−1.54 (14)
O16—Mo4—Mo5—O10	−2.8 (2)	O20—Co1—O19—Mo5	97.16 (14)
O16—Mo4—Mo5—O16	0.0 (2)	O20—Co1—O19—Mo6	1.53 (14)
O16—Mo4—Mo5—O17	148.9 (2)	O19—Co1—O21—Mo1	−84.05 (14)
O16—Mo4—Mo5—O19	85.2 (2)	O19—Co1—O21—Mo2	−179.76 (14)
O16—Mo4—Mo5—O24	149.5 (2)	O21—Co1—O19—Mo5	−179.00 (14)
Mo5—Mo4—O23—Mo3	−146.22 (9)	O21—Co1—O19—Mo6	85.36 (14)
Mo5—Mo4—O23—Co1	−40.60 (12)	O19—Co1—O23—Mo3	179.18 (14)
O23—Mo4—Mo5—O9	161.34 (8)	O19—Co1—O23—Mo4	81.48 (15)
O23—Mo4—Mo5—O10	−88.70 (9)	O23—Co1—O19—Mo5	−82.65 (15)
O23—Mo4—Mo5—O16	−85.91 (8)	O23—Co1—O19—Mo6	−178.29 (14)
O23—Mo4—Mo5—O17	63.03 (8)	O19—Co1—O24—Mo4	−95.67 (15)
O23—Mo4—Mo5—O19	−0.68 (8)	O19—Co1—O24—Mo5	−0.53 (14)
O23—Mo4—Mo5—O24	63.62 (8)	O24—Co1—O19—Mo5	0.53 (14)
Mo5—Mo4—O24—Mo5	0.0	O24—Co1—O19—Mo6	−95.11 (15)
Mo5—Mo4—O24—Co1	104.66 (16)	O20—Co1—O21—Mo1	−0.49 (13)
O24—Mo4—Mo5—O9	97.71 (12)	O20—Co1—O21—Mo2	−96.20 (14)
O24—Mo4—Mo5—O10	−152.32 (12)	O21—Co1—O20—Mo1	0.50 (14)
O24—Mo4—Mo5—O16	−149.54 (12)	O21—Co1—O20—Mo6	−97.44 (14)
O24—Mo4—Mo5—O17	−0.59 (12)	O20—Co1—O22—Mo2	84.29 (14)
O24—Mo4—Mo5—O19	−64.30 (12)	O20—Co1—O22—Mo3	−179.08 (14)
O24—Mo4—Mo5—O24	0.00 (12)	O22—Co1—O20—Mo1	−83.68 (14)
O7—Mo4—O8—Rb1^v^	−133.0 (5)	O22—Co1—O20—Mo6	178.39 (14)
O7—Mo4—O15—Mo3	−70.0 (2)	O20—Co1—O24—Mo4	−179.13 (14)
O7—Mo4—O16—Mo5	−176.6 (2)	O20—Co1—O24—Mo5	−83.98 (14)
O7—Mo4—O23—Mo3	82.94 (16)	O24—Co1—O20—Mo1	179.91 (14)
O7—Mo4—O23—Co1	−171.43 (17)	O24—Co1—O20—Mo6	81.97 (14)
O8—Mo4—O15—Mo3	−177.30 (19)	O21—Co1—O22—Mo2	0.39 (13)
O15—Mo4—O8—Rb1^v^	−32.2 (5)	O21—Co1—O22—Mo3	97.02 (14)
O8—Mo4—O16—Mo5	−67.8 (3)	O22—Co1—O21—Mo1	95.31 (14)
O16—Mo4—O8—Rb1^v^	123.6 (5)	O22—Co1—O21—Mo2	−0.39 (13)
O8—Mo4—O23—Mo3	−71.0 (4)	O21—Co1—O23—Mo3	−84.80 (15)
O8—Mo4—O23—Co1	34.7 (5)	O21—Co1—O23—Mo4	177.50 (14)
O23—Mo4—O8—Rb1^v^	19.8 (8)	O23—Co1—O21—Mo1	178.48 (14)
O8—Mo4—O24—Mo5	85.21 (15)	O23—Co1—O21—Mo2	82.78 (14)
O8—Mo4—O24—Co1	−170.13 (17)	O22—Co1—O23—Mo3	−0.74 (14)
O24—Mo4—O8—Rb1^v^	50.2 (5)	O22—Co1—O23—Mo4	−98.44 (15)
O15—Mo4—O16—Mo5	56.1 (4)	O23—Co1—O22—Mo2	−95.89 (15)
O16—Mo4—O15—Mo3	57.4 (4)	O23—Co1—O22—Mo3	0.74 (14)
O15—Mo4—O23—Mo3	−15.34 (12)	O22—Co1—O24—Mo4	84.96 (15)
O15—Mo4—O23—Co1	90.28 (16)	O22—Co1—O24—Mo5	−179.89 (14)
O23—Mo4—O15—Mo3	20.49 (15)	O24—Co1—O22—Mo2	−179.14 (14)
O15—Mo4—O24—Mo5	−179.88 (14)	O24—Co1—O22—Mo3	−82.51 (15)
O15—Mo4—O24—Co1	−75.22 (15)	O23—Co1—O24—Mo4	1.89 (14)
O24—Mo4—O15—Mo3	90.43 (17)	O23—Co1—O24—Mo5	97.04 (15)
O16—Mo4—O23—Mo3	−178.02 (14)	O24—Co1—O23—Mo3	95.80 (15)
O16—Mo4—O23—Co1	−72.40 (16)	O24—Co1—O23—Mo4	−1.90 (14)
O23—Mo4—O16—Mo5	91.14 (19)	O28—Co2—O32—Rb1	37.56 (13)
O16—Mo4—O24—Mo5	−16.17 (13)	O32—Co2—O28—Rb1	−35.90 (12)
O16—Mo4—O24—Co1	88.49 (17)	O32—Co2—O28—C4	−175.6 (3)
O24—Mo4—O16—Mo5	21.76 (17)	O33—Co2—O28—Rb1	−122.99 (13)
O23—Mo4—O24—Mo5	−106.37 (13)	O33—Co2—O28—C4	97.3 (3)
O23—Mo4—O24—Co1	−1.71 (13)	O34—Co2—O28—Rb1	140.73 (12)
O24—Mo4—O23—Mo3	−103.91 (13)	O34—Co2—O28—C4	1.0 (3)
O24—Mo4—O23—Co1	1.71 (13)	O28—Co2—O35—Rb1	−48.56 (12)
Mo4—Mo5—O16—Mo4	0.000 (14)	O35—Co2—O28—Rb1	48.91 (13)
Mo4—Mo5—O17—Mo6	−90.06 (18)	O35—Co2—O28—C4	−90.8 (3)
Mo4—Mo5—O19—Mo6	146.27 (9)	O33—Co2—O32—Rb1	121.10 (14)
Mo4—Mo5—O19—Co1	41.96 (12)	O32—Co2—O35—Rb1	46.77 (13)
Mo4—Mo5—O24—Mo4	0.0	O35—Co2—O32—Rb1	−49.60 (13)
Mo4—Mo5—O24—Co1	−104.43 (16)	O36—Co2—O32—Rb1	−148.82 (15)
O9—Mo5—O16—Mo4	69.6 (3)	O34—Co2—O35—Rb1	−140.10 (12)
O9—Mo5—O17—Mo6	176.3 (2)	O36—Co2—O35—Rb1	136.14 (13)
O9—Mo5—O24—Mo4	−83.39 (16)	Rb1—O28—C4—O29	−110.3 (5)
O9—Mo5—O24—Co1	172.19 (18)	Rb1—O28—C4—C5	71.0 (5)
O10—Mo5—O16—Mo4	177.8 (2)	Co2—O28—C4—O29	13.8 (7)
O10—Mo5—O17—Mo6	67.3 (3)	Co2—O28—C4—C5	−164.9 (3)
O10—Mo5—O19—Mo6	−86.24 (16)	O25—C1—C2—N1	165.7 (4)
O10—Mo5—O19—Co1	169.46 (18)	O25—C1—C2—C3	−71.9 (6)
O10—Mo5—O24—Mo4	77.3 (5)	O26—C1—C2—N1	−15.5 (6)
O10—Mo5—O24—Co1	−27.1 (5)	O26—C1—C2—C3	106.9 (5)
O16—Mo5—O17—Mo6	−58.1 (4)	N1—C2—C3—O27	61.8 (6)
O17—Mo5—O16—Mo4	−55.4 (4)	C1—C2—C3—O27	−60.2 (6)
O16—Mo5—O19—Mo6	177.87 (14)	O28—C4—C5—N2	6.4 (6)
O16—Mo5—O19—Co1	73.57 (16)	O28—C4—C5—C6	129.8 (4)
O19—Mo5—O16—Mo4	−90.95 (19)	O29—C4—C5—N2	−172.4 (4)
O16—Mo5—O24—Mo4	16.10 (13)	O29—C4—C5—C6	−49.0 (6)
O16—Mo5—O24—Co1	−88.33 (17)	N2—C5—C6—O30	66.2 (5)
O24—Mo5—O16—Mo4	−21.67 (17)	C4—C5—C6—O30	−57.3 (6)
O17—Mo5—O19—Mo6	15.87 (12)		

Symmetry codes: (i) −*x*+1, *y*−1/2, −*z*+1/2; (ii) −*x*+2, *y*−1/2, −*z*+1/2; (iii) −*x*+2, *y*+1/2, −*z*+1/2; (iv) *x*+1, *y*, *z*; (v) −*x*+1, *y*+1/2, −*z*+1/2; (vi) *x*, *y*+1, *z*; (vii) *x*+1/2, −*y*+1/2, −*z*; (viii) *x*+1/2, −*y*+3/2, −*z*; (ix) *x*−1/2, −*y*+1/2, −*z*; (x) *x*−1, *y*, *z*; (xi) −*x*+3/2, −*y*+1, *z*+1/2; (xii) *x*−1/2, −*y*+3/2, −*z*; (xiii) −*x*+3/2, −*y*+1, *z*−1/2; (xiv) *x*+1, *y*−1, *z*; (xv) *x*+1, *y*+1, *z*; (xvi) *x*−1, *y*+1, *z*; (xvii) *x*, *y*−1, *z*; (xviii) *x*−1, *y*−1, *z*.

### Hydrogen-bond geometry (Å, º)

**Table:**

*D*—H···*A*	*D*—H	H···*A*	*D*···*A*	*D*—H···*A*
O27—H4···O41	0.84	2.24	2.859 (6)	131
O27—H4···N1	0.84	2.62	2.940 (6)	104
O30—H11···O40	0.84	2.56	2.708 (6)	91
O30—H11···O42^vii^	0.84	2.24	2.928 (6)	139
N1—H5···O2	0.91	2.11	2.944 (6)	151
N1—H6···O26	0.91	2.49	2.647 (6)	89
N1—H6···O39^vii^	0.91	2.14	3.041 (6)	170
N1—H7···O17^ii^	0.91	2.09	2.905 (6)	149
N2—H13···O28	0.91	2.60	2.691 (6)	86
N2—H13···O43	0.91	2.11	3.013 (7)	170
N2—H12···O8^i^	0.91	2.31	2.912 (6)	123
N2—H12···O28	0.91	2.61	2.691 (6)	85
N2—H14···O13^xvii^	0.91	1.93	2.827 (6)	167

Symmetry codes: (i) −*x*+1, *y*−1/2, −*z*+1/2; (ii) −*x*+2, *y*−1/2, −*z*+1/2; (vii) *x*+1/2, −*y*+1/2, −*z*; (xvii) *x*, *y*−1, *z*.
